# The sobol sensitivity analysis of the pressure, stresses, and displacement arising from poroelastic modelling of hard mechanical systems

**DOI:** 10.1038/s41598-025-28109-z

**Published:** 2025-12-23

**Authors:** Hadi Asghari, Laura Miller, Raimondo Penta, Andrey Melnikov, Christos Spitas, Jose Merodio

**Affiliations:** 1 Department of Mathematics, School of Sciences and Humanities, Nazarbayev University, Kabanbay Batyr 53, 010000 Astana, Kazakhstan; 2 Department of Mathematics and Statistics, University of Strathclyde, Livingstone Tower, 26 Richmond Street, G1 1XH Glasgow, UK; 3 School of Mathematics and Statistics, University of Glasgow, G12 8QQ Glasgow, UK; 4 Department of Mechanical, Materials and Manufacturing Engineering, University of Nottingham, Ningbo, China; 5 Departamento de Matematica Aplicada a las TIC, Universidad Politecnica de Madrid, Madrid, Spain; 6 Institute of Mathematics, Faculty of Technical Physics, Information Technology and Applied Mathematics, Lodz University of Technology, Lodz, Poland

**Keywords:** Sobol statistical method, Biot’s poroelasticity, Hard mechanical systems, Energy science and technology, Engineering, Materials science, Solid Earth sciences

## Abstract

We perform a sensitivity analysis to investigate the influence of material input parameters on the pressure, stresses, and displacement of an isotropic porous solid cylinder representing hard mechanical systems such as the bone. We model the system using the governing equations of Biot’s poroelasticity in cylindrical polar coordinates, where the solutions are found by enforcing radial boundary conditions. The sensitivity analysis is carried out on the solutions for the pressure, stress components and displacement using ranges of the investigated parameters representative of the bone. Our study finds that the time $t^*$ has the highest influence on the pressure, the stress components and displacements. We find that the Poisson ratio $\nu$ plays a greater role than shear $\mu$ in the pressure response, and the shear $\mu$ counts more than the other parameters in the radial and circumferential stresses. There are key joint interactions between the Biot’s coefficient $\alpha$ and the Poisson ratio $\nu$, the non-dimensionalised radius $R^*$ of the bone, and the Biot’s modulus *M* when investigating interstitial pressure, which is a key value in bone remodelling and fracture healing. This study paves the way to a deeper understanding of the interplay of all the parameters that are necessary to capture the true behaviour of hard mechanical systems such as the bone and its potential remodelling.

## Introduction

The Theory of Poroelasticity describes the effective mechanical behaviour of fluid-saturated porous elastic materials. Its development was grounded in experimental observations and established through early foundational studies^1–4^. The theory can be used to describe the behaviour in physical situations where the interactions between the fluid and the deformable solid occur on the porescale. Among the various situations in which the method has been used is the modelling of rigid, hierarchical tissues such as bones^5,6^. The interstitial matrix of biological tissues, which can be either healthy or tumorous^7,8^, as well as soft biological tissues like the heart (myocardium) and artery walls^9–12^ can also be modelled using this theory. Outside of animal biology, the theory is especially applied to model hard mechanical systems, such as soil, rocks^13,14^, as well as artificial structures utilised for biomimetic materials and regenerative therapies^15,16^, and the behaviour of hard tissues such as those present in the bones.

The microstructure of human bones is complex and uniquely designed to give the correct strength, flexibility, and biological adaptation. Bones consist of cortical bone, which forms the dense outer shell, and trabecular (spongy) bone, a porous, lattice-like structure found inside. The cortical bone is composed of osteons, cylindrical units containing Haversian canals with blood vessels and nerves, ensuring nutrient flow^17^. Trabecular bone, found at the ends of long bones and within vertebrae, distributes mechanical stress efficiently while maintaining lightweight strength. The bone matrix is rich in collagen fibres, which provide flexibility, and hydroxyapatite crystals, a mineral compound that gives bones their rigidity^18^. Further details regarding bone microstructure can be found in the literature^6,19–22^.

The isotropic porous cylinder considered in this work represents bones. Previous research has examined the use of porous media for bone modelling^5,23,24^. Bone marrow, blood, or interstitial fluid and cells fill the pores of the porous solid that describes the bone^24,25^. Osteocytes, which are mechanosensitive cells present in the pore interstitial fluid, regulate bone maintenance, while continuous remodelling by osteoblasts (bone-forming cells) and osteoclasts (bone-resorbing cells) keeps bones healthy and resilient. The osteoblasts and osteoclasts also regulate the process by which bones remodel in response to stress, damage, or growth^24^. This intricate structure allows bones to withstand forces while adapting to physiological demands.

According to Soleimani et al. (2024), current research has demonstrated that the pressure and velocity of the interstitial fluid passing through the bones’ pores significantly influences the process of bone remodelling^26–28^. It has also been added that the interstitial fluid velocity and the shear stress inside the bones may have an impact on the cells that influence bone healing^27^.

Research into poroelastic modelling of bone has advanced significantly via both micromechanical and numerical approaches. Swan et al. developed a micromechanical poroelastic model of cortical bone, using unit-cell analysis to capture fluid flow within Haversian systems and linking microstructure to effective poroelastic properties^29^. Their work demonstrated how mechanical loading induces fluid shear stresses that may influence bone adaptation. More recently, multiscale homogenisation techniques have been applied to model bone as a poroelastic medium, accounting for its hierarchical structure and fluid-saturated nature^24^. Finite element methods have been widely used to simulate bone mechanics, with stabilized formulations enabling accurate modelling of nonlinear poroelastic behaviour^30,31^. These FE-based models have been instrumental in predicting stress-strain responses and exploring mechanotransduction pathways in bone tissue.

It is possible to solve the governing equations of poroelastic materials analytically or numerically in certain situations. Numerous parameters are involved in the solutions that can be found. The overall pressure, stress, or displacement (the solution) will be affected differently by each of the (input) parameters. A sensitivity analysis can be performed to ascertain the significance of each parameter and how the interactions between the various input parameters impact the solution output.

Sensitivity analyses are implemented to grasp the impact of the different input values on the model behaviour, and can have a significant role in different areas of model development such as model substantiation, research prioritization, model improvement, and model verification^32^. Moreover, sensitivity analysis gives a comprehensive overview of how the interactions between the different input parameters affects the behaviour of the model output^33^. Recently, various areas of engineering and science that investigate complex models with industrial biomechanical applications have utilized sensitivity analysis techniques (see, Anstett-Collin et al.^34^, and Saisana et al.^35^). This led to the application of sensitivity analysis to study the inflation of tubular structures^36–39^ and closely related problems^34,35,40–42^. To obtain a thorough understanding of the mechanical behaviour of a material it is important to quantify and qualify the degree of significance of the input parameters as well as their contributions to the output of the model. Sensitivity analysis addresses these points, in particular, it evaluates the behaviour of a model focusing on how the input parameters affect and interact with the output variables.

There are several categorizations for sensitivity analysis methods. One of the most prominent is proposed by Frey & Patil^43^. They segment the sensitivity analysis methods into three groups: a) the mathematical approach that encompasses the automatic differentiation, the nominal range, log-odds ratio and the break-even analysis; b) the graphical approach that entails the visualization tools such as scatter plots and heat maps and; c) the statistical approach that includes probabilistic models with simulation methods and corresponding estimators. The Sobol method, the Fourier amplitude sensitivity test (FAST) method, the regression analysis, and the Morris method are defined in the framework of statistical sensitivity analysis methods. Some of these methods are applied under some constraints, for example, the Morris and regression methods, can only be applied to monotonic models, while the Sobol and FAST methods can be implemented in complex problems with non-monotonic and non-linear behaviour. Recent works applied the Sobol and the FAST methods to study the extension, inflation, and torsion of a circular cylindrical tube in the presence of residual stresses^36,44^ .

Within this work we investigate a solid cylinder whose microstructure consists of a porous elastic matrix. The elastic matrix of the solid cylinder is assumed to be isotropic. Bones experience a complex interplay of pressure, stress, and displacement due to various factors, leading to deformation, microscopic damage as well as bone loss and fracture. It is important to unravel the effect that the input variables have on these mechanisms and the first step is to understand precisely pressure, stress, and displacement in bones. We begin by summarising the analytic solution to the governing equations of a poroelastic material in cylindrical polar coordinates subject to radial boundary conditions. We use the poroelastic equations and the corresponding solution (pressure, stresses and displacement) to model the behaviour of bones. In particular, to better understand the behaviour of bones, we investigate the solutions for pressure, stresses and displacement via a Sobol sensitivity analysis. This analysis allows us to determine which one of the input poroelastic parameters has the greatest influence on the pressure, stresses and displacement of bones, as well as which interactions among (input) parameters are important, and can provide understanding of bone strength and fracture healing.

The paper is organized as follows. In Sect. 2 we summarise the analytical solution for the equations that govern a porous elastic cylinder representing bones. In Sect. 2.1 we investigate the series solutions in order to understand how to simplify them prior to the sensitivity analysis. In Sect. 3 we summarise the key and important features of the sensitivity analysis technique and discuss the approach we will take to investigate the parameters that influence stress, displacement and pressure in a porous elastic isotropic cylinder representing bone. In Sect. 4 we apply a Sobol sensitivity analysis to the expanded and simplified pressure, stress and displacement equations to understand the role that each of the parameters has on the resulting behaviour of the material. Finally in Sect. 5 we provide concluding remarks and future perspectives for this work.

## Summary of the analytical solution for a poroelastic cylinder

In this section, we relate the bone microstructure to a porous cylinder to facilitate our modelling approach. We then summarise the analytical solution for the equations governing an isotropic porous elastic cylinder that was obtained in Asghari et al. (2024)^37^.

**Fig. 1:**
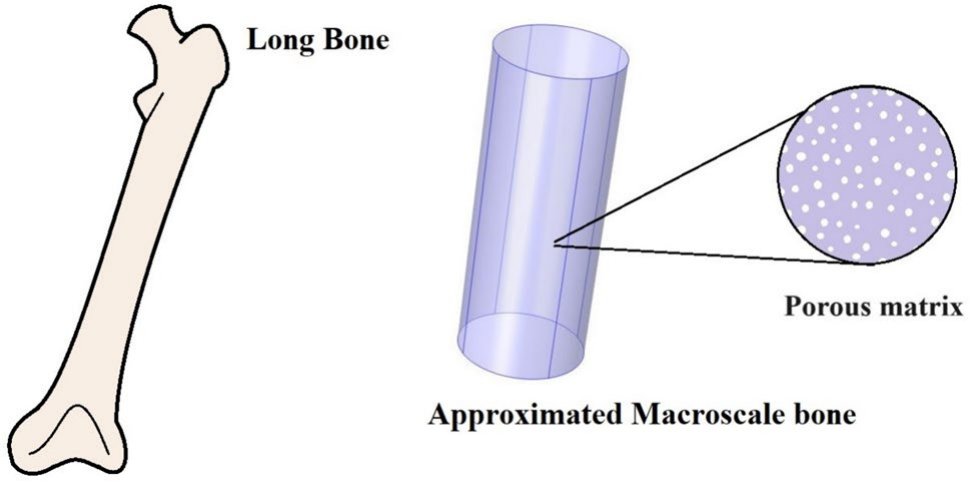
A sketch of a human long bone which we approximate as a cylinder with an underlying porous microstructure.

Bone is a porous, fluid-saturated material which contains a solid matrix of collagen and mineral (hydroxyapatite) filled with interstitial fluid. Poroelasticity captures the interaction between mechanical deformation and fluid flow, which is crucial for load transmission, nutrient transport, and mechanotransduction within the bones. Long bones such as those in human limbs (e.g., femur, tibia) are elongated structures with an approximately cylindrical shape, especially in the shaft (as shown in Fig. 1). The cortical bone forms a hollow cylinder around the medullary cavity, making the cylindrical approximation geometrically reasonable. The cortical bone (outer shell) bears the majority of mechanical loads in long bones. The medullary cavity is filled with marrow, which has low stiffness and minimal structural contribution under typical loading conditions. For our model which is focused on pressure, stresses and displacement, the cavity’s influence is negligible. The medullary cavity plays a role in creation of new blood cells and metabolic activity, but not in mechanical stiffness or load transmission and therefore we are permitted to neglect. This simplification allows for standard poroelasticity in cylindrical coordinates to be used to model the bone.

We are now ready to consider the bone as a solid porous elastic cylinder. We consider the case of radial inflation. We can write the displacement field for this cylinder (bone) in cylindrical polar coordinates, assuming axisymmetry and plane strain, as

1 $$\begin{aligned} \textbf{u}=u_r\textbf{e}_r+0\textbf{e}_{\theta }+0\textbf{e}_z=(u(r), 0,0). \end{aligned}$$

The equilibrium equation (with axial symmetry) can be written as

2 $$\begin{aligned} \frac{\partial \sigma _{rr}}{\partial r}+\frac{\sigma _{rr}-\sigma _{\theta \theta }}{r}=0, \end{aligned}$$

in terms of the (radial and azimuthal) components of the stress tensor $\varvec{\sigma }$. The stress of the material can be written as

3 $$\begin{aligned} \varvec{\sigma }=\lambda \textrm{tr}(\textbf{e})\textsf{I} + 2\mu \textbf{e}-\alpha M \zeta {\mathsf I}, \quad \text{ where } \quad \textbf{e} {(\textbf{u})}=\frac{\nabla \textbf{u} +(\nabla \textbf{u})^{\textrm{T}}}{2}, \end{aligned}$$

is the strain, tr is the trace, $\lambda$ is the Lamé constant, which can be written in terms of shear $\mu$ and Poisson ratio $\nu$ as $\lambda =\frac{2\mu \nu }{1-2\nu }$, $\alpha$ is the Biot’s coefficient, *M* is the Biot’s modulus and $\zeta$ is the fluid content. The equation governing the pressure reads

4 $$\begin{aligned} P=M(\zeta -\alpha \textrm{tr}(\textbf{e})). \end{aligned}$$

In order to solve equations (2), with (3), and (4), we perform a Laplace transform to the equations and impose radial stress boundary conditions to find the solution in the Laplace transformed domain. In summary, the inner surface of the cylinder is subjected to a uniform radial stress, $\sigma _{rr}=-P_0$ and the pore pressure is drained, $P=0$, i.e. the boundary conditions are $\sigma _{rr}=-P_0; \quad P=0; \quad \text{at}\quad r=r_0.$

One then needs to revert the solution back to the time domain via an inverse Laplace transformation. Full details of this process are presented by Asghari et al. (2024)^37^.

The (final) solution for the pressure, stresses and displacement is

5 $$\begin{aligned}&\frac{P}{P_0}=\sum _{n=1}^\infty \bigg (\frac{2\alpha {x_n}^{3/2}(1-2\nu )[2\mu (1-\nu )(J_0(R^*\sqrt{x_n}){{-}}MJ_0(\sqrt{x_n}))-\alpha ^2M(1-2\nu )(J_0(R^*\sqrt{x_n})-M J_0(\sqrt{x_n}))] e^{-x_nt^*}}{\mu C(x_n)}\bigg )\end{aligned}$$

6 $$\begin{aligned}&\frac{\sigma _{rr}}{P_0}=\sum _{n=1}^\infty \bigg (\frac{-2x_n [\alpha ^2 M(1-2\nu )[2(1-2\nu )J_1(R^*\sqrt{x_n})-\sqrt{x_n}R^*J_0(\sqrt{x_n})]+2\mu \sqrt{x_n}R^*(1-\nu ) J_0(\sqrt{x_n})]e^{-x_nt^*}}{R^* C(x_n)}\bigg )\end{aligned}$$

7 $$\begin{aligned}&\frac{\sigma _{\theta \theta }}{P_0}=\sum _{n=1}^\infty \bigg (\frac{-2x_n[\alpha ^2 M(1-2\nu )[2(1-2\nu )[J_1(R^*\sqrt{x_n})\sqrt{x_n}R^*J_0(R^*\sqrt{x_n})]-\sqrt{x_n}R^*J_0(\sqrt{x_n})] +2\mu \sqrt{x_n}R^*(1-\nu )J_0(\sqrt{x_n})]e^{-x_nt^*}}{R^* C(x_n)}\bigg )\end{aligned}$$

8 $$\begin{aligned}&\frac{\sigma _{zz}}{P_0}=\sum _{n=1}^\infty \bigg (\frac{4{x_n}^{3/2}[\alpha ^2 M(1-2\nu )[(1-2\nu )J_0(R^*\sqrt{x_n})+\nu J_0(\sqrt{x_n})]-2\nu \mu (1-\nu )J_0(\sqrt{x_n})]e^{-x_nt^*}}{C(x_n)}\bigg )\end{aligned}$$

9 $$\begin{aligned}&\frac{u_{r}}{r_0}=\sum _{n=1}^\infty \bigg (\frac{-x_n P_0 r_0(1-2\nu )[\alpha ^2 M(1-2\nu )[2J_1(R^*\sqrt{x_n})-R^*\sqrt{x_n}J_0(\sqrt{x_n})]+2\mu (1-\nu )R^*\sqrt{x_n}J_0(\sqrt{x_n})]e^{-x_nt^*}}{\mu C(x_n)}\bigg ) \end{aligned}$$

respectively, where

10 $$\begin{aligned} C(x_n)=(2(1-\nu )\mu -\alpha ^2M(1-2\nu ))[J_0(\sqrt{x_n})\sqrt{x_n}-x_nJ_1(\sqrt{x_n})]-2\alpha ^2M(1-2\nu )^2[J_0(\sqrt{x_n})\sqrt{x_n}-J_1(\sqrt{x_n})] \end{aligned}$$

$x_n$ are real and positive, $J_0$ and $J_1$ are the Bessel functions of the first kind of zero and first-order, respectively, and $t^*$ ($t^*=\frac{ct}{r_0^2}$, where c is the consolidation coefficient) and $R^*$ ($R^*=\frac{r}{r_0}$) are the non-dimensionalised time and radius of our cylinder, respectively. This is the full analytical solution for the pressure, stresses and elastic displacement of an isotropic porous cylinder.

### Simplification of the solutions prior to the sensitivity analysis

**Fig. 2:**
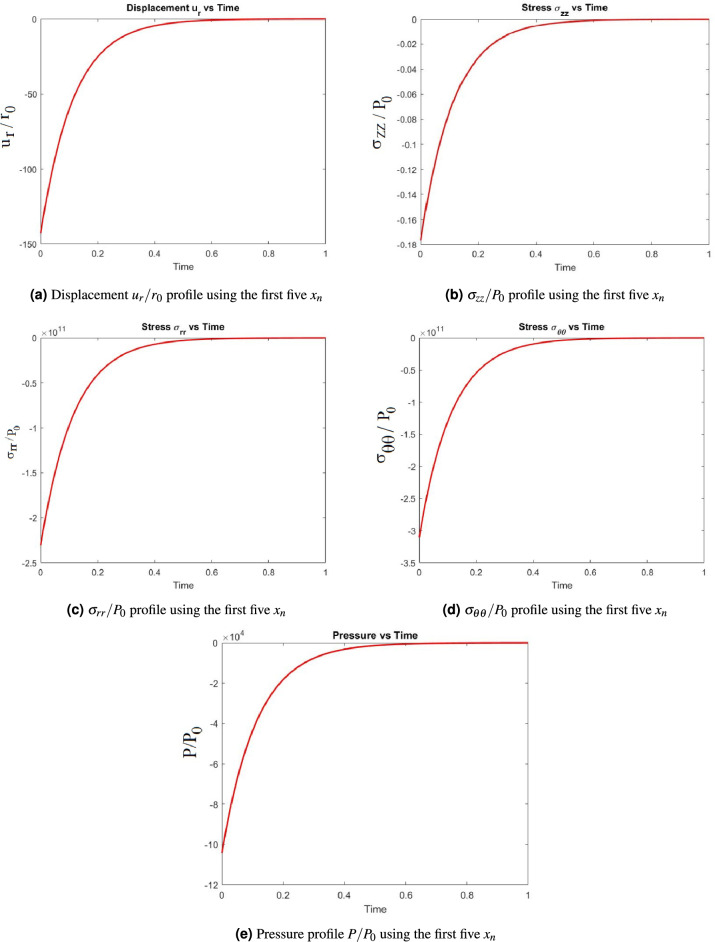
Solution profiles for the porous cylinder using the first five $x_n$.

In this work we will focus on the sensitivity analysis of the parameters appearing in the solutions of pressure, stress components, and displacement (5)-(9). Each of these expressions is a sum evaluated for $x_n$, $n=1,...,\infty$. The $x_n$ are the zeros of the denominator (10) of the solutions (5)-(9). The expanded version of these sums can be extremely lengthy expressions, making it difficult to carry out the sensitivity analysis. Therefore one needs to simplify the sums for the pressure, stresses and displacements without losing key information. We do this by considering only the first five terms of each of the sums (5)-(9). This allows us to have much shorter expressions. Results are illustrated in plots Fig. 2a–e for the non-dimensionalised pressure, displacement and stresses and show on the one hand that the solutions converge and on the other hand that no information is being lost truncating the series sum by taking just 5 zeros. The procedure is the following. To expand the sum, we need to calculate the first five zeros of the denominator of the solutions in the Laplace transformed domain, where we assume that the parameters are not varying. This denominator is given by

11 $$\begin{aligned}&\mu I_0(\sqrt{S^*})(2\sqrt{S^*}(1-\nu )\mu -\alpha ^2M(1-2\nu )\sqrt{S^*})-2\mu \alpha ^2M(1-2\nu )^2I_1(\sqrt{S^*})=0, \end{aligned}$$

where $I_i$, $i=0,1$ is the modified Bessel function of the first kind of order *i*, $S^*=\frac{r_0^2s}{c}$ with s being the transformation variable during Laplace transformation, and in which one needs to fix the (input) parameters to determine the $x_n$. Full details of the steps involved to obtain (11) are given in Asghari et al. (2024)^37^. We are considering the standard poroelastic parameters: shear and Poisson ratio, $\mu$ and $\nu$, as well as Biot’s modulus and coefficient, *M* and $\alpha$. The shear modulus $\mu$ and Biot’s modulus *M* have the units of pressure (GPa/KPa). The Poisson ratio $\nu$ and Biot’s coefficient $\alpha$ are non-dimensional. Further details on the standard poroelastic coefficients can be found in the literature^45,46^.

We are trying to model an intrinsically incompressible porous material. In porous media, the Poisson ratio $\nu$ is constrained to the region between 0 and 0.5, where when $\nu =0$ the material is fully compressible and when $\nu =0.5$ it is fully incompressible. If the Biot’s modulus *M* is approaching infinity, together with Biot’s coefficient $\alpha$ approaching 1, then, this represents a situation where the material will exhibit intrinsic incompressibility.

We choose $\mu =1$; $\nu =0$; $\alpha =1$ and $M=50000$ and solve (11) to obtain the first 5 $x_n$, which are:

12 $$\begin{aligned} x_n=[8.7002, 34.119, 78.726, 142.95, 226.88]. \end{aligned}$$

Since there is a smooth profile for all solutions shown in Fig. 2a–e one can deduce that is sufficient to determine the behaviour of stresses, displacement and pressure using the first 5 $x_n$ in each sum. We are now able to carry out the sensitivity analysis for the (input) parameters of (5)- (9) using the first 5 $x_n$.

## The sensitivity analysis

Sensitivity analysis investigates how variations in input parameters influence the output of the model. Indeed, it it very important to know how each input parameter and its associated uncertainty variability affects the output of the model. Specifically, if a probability distribution is assigned to each input parameter in a model, then, sensitivity analysis methods can be used to understand the parameters influence on the model output. Recently, different sensitivity analysis methods have been applied to some mechanical problems such as extension, inflation, and torsion of a circular cylindrical tube^36^. For instance, the Fourier amplitude sensitivity test method and the Sobol method have been applied on the inflated cylindrical tube^44^, on an inflated and extended fibre-reinforced membrane with different natural configurations of its constituents^39^ as well as on fibrous thick-walled tubes with mechano-sensitive remodelling fibres in homeostasis^38^, and on an isotropic porous solid cylinder^37^.

Here, we deploy one of the most applicable statistical methods among sensitivity analysis techniques, namely the Sobol method, due to the non-linear and non-monotonic characteristics of the problem at hand. In what follows, we summarise the Sobol method in Section 3.1 and then discuss the applied probability distributions that we use with the Sobol method (uniform, exponential, and Weibull distributions) in Section 3.2. Finally, in Section 3.3, we summarise the measurements of assessment that we use: the bias measure and the standard deviation.

### Sobol method

The Sobol method is categorized as a *variance-based sensitivity analysis* (VBSA) and as a numerical method^32^. It can be considered as a statistical approach within sensitivity analysis methods. It quantifies the contribution of each input parameter to the variability of the model outputs, thereby revealing the primary sources of uncertainty within the system^33^. By applying this method, we assess the extent to which individual input parameters and their interactions influence the variability of the model’s output^47^.

Consider that $X=(X_1,\ ...,\ X_e)$ is a random vector and that $y=g(X)$. In addition, $g(\cdot )$ is a function that is defined on the interval ${[0,\ 1]}^e$ as,

13 $$\begin{aligned} \begin{array}{lllllllllll} g\left( X\right) = g_0+\sum ^e_{i=1}{g_i\left( X_i\right) } +\sum ^e_{i<j}{g_{ij}(X_i,X_j)}+\dots +g_{12\dots e}(X), \end{array} \end{aligned}$$

where

14 $$\begin{aligned} \begin{array}{llllll} \displaystyle \int ^1_0{g_{i_1\dots i_k}\left( X_{i_1},\dots ,X_{i_k}\right) dx_{i_l}=0},\qquad 1\le l\le k,\\ \displaystyle \{i_1,\ ...,i_k\} \subseteq \{1,...,e\}, \end{array} \end{aligned}$$

with *e* the number of input variables^33^. The functional *analysis of variance* (ANOVA) is shown in (15) and it is the variance of a function *y*, and can be written as

15 $$\begin{aligned} \textrm{Var}\left( y\right) =\ \sum ^e_{i=1}{D_i\left( y\right) }+\sum ^e_{i<j}{D_{ij}(y)}+\dots +D_{12\dots e}(y), \end{aligned}$$

where $\textrm{Var}[E(y\ |X_i)]=D_i(y)$ are first-order indices, *E* is the mathematical expectation for conditional probability of *y* when $X_i$ and $X_j$ are specified in the model, the second-order indices are shown as $D_{ij}(y)=\textrm{Var}[E(y\ |X_i,\ X_j)]-D_i(y)-D_j(y)$, etc^48^. The first-order indices and second-order interaction indices are given, respectively, as,

16 $$\begin{aligned} S_i=\dfrac{D_i(y)}{\textrm{Var}(y)},\qquad S_{ij} =\dfrac{D_{ij}(y)}{\textrm{Var}(y)}, \end{aligned}$$

where $2^e-1$ indices are available in this method. For instance, if one considers five input variables, i.e., $e = 5$, then, there are $2^5-1 = 31$ indices, which include individual and interaction effects in the model^49^. The following total indices are proposed by Archer et al.^50^:

17 $$\begin{aligned} S_{T_i}=S_i+\sum _{i<j}{S_{ij}}+\sum _{j\ne i,l\ne i,j<l}{S_{ijl}}+\dots =\sum _{l\in \#i}{S_l}, \end{aligned}$$

where $\#i$ refers to all the subsets of $\{1,\ ...,\ e\}$ that include the index *i*.

To obtain variance-based sensitivity indices, it is necessary to specify a sampling design and an estimator. With regard to the sampling design, Sobol^51^ proposed the Monte-Carlo sampling-based method for first-order and interaction indices. Later on, Saltelli^49^ developed this method for both first-order and total indices. By means of random repetition^52^, bootstrap methods^50^ and asymptotic formulas^53^ in the Monte-Carlo method, the error estimation can be obtained for the indices, which is an advantage of this method.

Regarding the selection of the (best) estimator, some researchers have proposed practical and suitable estimators for Sobol indices. Here, for first-order effect, the *“Saltelli estimator”*^54^ that is proposed by Saltelli et al. is considered while for total sensitivity indices, the *“Janson estimator”*^55^ given by Puy et al.^56^ is taken into account. Based on previous similar problems, these estimators are suitable for non-monotonic and non-linear models.

The Saltelli estimator is expressed for first-order indices as

18 $$\begin{aligned} S_i=\displaystyle \frac{\displaystyle \frac{1}{e}\sum ^e_{j=1}g\left( B\right) _j \left[ g\left( A^{\left( i\right) }_B\right) _j-{g\left( A\right) }_j\right] }{\textrm{Var}(y)}, \end{aligned}$$

which is composed of combinations of the matrices $A,\ B,$ $A^{\left( i\right) }_B$ or  $B^{\left( i\right) }_A$, The full expression is given in Puy et al.^56^.

The Janson estimator for the total indices is

19 $$\begin{aligned} T_i=\frac{\displaystyle \frac{1}{2e}\sum ^e_{j=1}\left[ g\left( A\right) _j-{g\left( A^{\left( i\right) }_B\right) }_j\right] ^2}{\textrm{Var}(y)}. \end{aligned}$$

To obtain these estimators, the sampling-based approach is used. The joint effect of an input using Sobol indices is given by $T_i-S_i$. In particular, the uncertainty of the model output is shown by the joint effects of the input parameters $X_i$ with the other input parameters. If $X_i$ does not have any total effect in the model, then $T_i =0$, and one can conclude that it is not an influential input in the model (it can be taken as a fixed constant)^55^.

Second-order interactions, given in Eq. (16) depict the interaction effect between two input parameters on the model output. The estimations of second-order interactions are given by means of Liu and Owen’s formula as

20 $$\begin{aligned} \begin{array}{llllllllllll} \hat{S}_{i,j}=\displaystyle \dfrac{1}{4n} \sum ^n_{k=1}(g(X_i^k,X_j^k,X^k_{-{i,j}} ) -g(X_i^k,W_j^t,X^k_{-{i,j}} )-g(W_i^k,X_j^k,X^k_{-{i,j}}) +g(W_i^k,W_j^k,X^k_{-{i,j}}))^2, \end{array} \end{aligned}$$

where $W_i$ and $W_j$ are the two independent copies expressed on the interval ${[0,\ 1]}^e$ from $X_i$ and $X_j$, respectively, *k* is the number of repetitions in model output, and *n* is the number of the sample size of input parameters^57^. In addition, $X_{-{i,j}}$ depicts the higher-order interaction effects of two involved input parameters without the individual effect of the *i*, *j* parameters in the model. In other words, Eq. (20) represents the mathematical expectation of the interaction effects among input parameters on the model output. These interaction effects are visualised through a multi-scatter plot matrix, which illustrates the relationships between input parameters and output variables. Specifically, scatter plots depicting the influence of input parameters on pressure, displacement, and stress components are presented in Section 4, providing insight into the joint contributions and dependencies within the poroelastic bone model.

We consider three probability distributions for the input parameters instead of assigning them random data or deterministic fixed data. This is convenient to run statistical simulations in “R” programming using the Sobol method. In particular, the uniform, exponential, and Weibull probability distributions are applied by means of *“Saltelli-Jansen”* estimators (18) and (19) to obtain the Sobol indices of the input parameters.

### The probability distributions

The probability distribution function characterises all possible values that a random variable may assume^58^. Bone properties vary significantly depending on factors such as anatomical location, age, and physiological condition. Consequently, parameters associated with bone-when modelled as a porous solid, such as Biot’s modulus-are distributed across specific physical ranges that directly influence output variables like pressure, stress components, and displacement. This implies that the model’s results are sensitive to the choice of input parameter distributions. In our study, the structural behaviour of an isotropic porous cylindrical bone is governed by parameters including time, bone radius, Poisson’s ratio, and Biot’s modulus. To account for variability, we consider uniform, exponential, and Weibull distributions for the input parameters, with each distribution defined over a range consistent with its physical domain.

#### Uniform distribution

When all events have the same chance of occurrence, one can associate a continuous uniform distribution. In this distribution, two scalar quantities associated with a variable, say *X*, are defined: a minimum value *p*, and a maximum value *q*. For the uniform distribution, the probability density function is as follows:^32^

21 $$\begin{aligned} f(X) = \left\{ \begin{array}{llllll} \displaystyle \frac{1}{q-p} & \quad \text{ for }\quad & p< X< q, \\ 0 & \quad \text{ for }\quad & X<p\quad \text{ or } \quad X>q. \end{array} \right. \end{aligned}$$

The histogram of equation (21) is a *rectangular distribution function*. The uniform distribution of a variable *X* is denoted as $X \,{\sim }\, \mathscr {U}(p,q)$.

#### Exponential distribution

The exponential distribution is another continuous probability distribution, which considers the values of a parameter until a certain event occurs. One property of the exponential distribution is its lack of memory, i.e., previous actions of a parameter do not impact future actions^58^. This type of probability distribution has many applications in real life such as medicine, economics, and engineering. A random variable, *X*, follows the exponential distribution with the following probability density function:

22 $$\begin{aligned} f(X) = \left\{ \begin{array}{llllll} \displaystyle (1/ \beta ) e^{-(1/ \beta ) X} & \quad \text{ for }\quad & X > 0, \\ 0 & \quad \text{ for }\quad & X\le 0.\quad \end{array} \right. \end{aligned}$$

The exponential distribution of a variable *X* is denoted as $X \,{\sim }\,{\textrm{exp}}(1/ \beta )$.

#### Weibull distribution

In the modelling of lifetime problems, the Weibull distribution is commonly used. The Weibull distribution is categorized as a continuous probability distribution. It is used for modelling failure time and for time between events, for example, the maximum one-day snowfall or the time a group of teenagers surf the web, both follow the Weibull distribution. The distribution formula of Weibull is

23 $$\begin{aligned} f(X)= \theta \alpha (\theta X)^{\alpha -1}e^{-(\theta X)^\alpha } \quad \text{ for }\quad X > 0. \end{aligned}$$

The Weibull distribution is applied in engineering fields, such as modelling stochastic processes related to the time of manufacturing, radar systems, and to simulate the dispersion of the received signal.

In this work, we assume the scale parameter of the Weibull distribution to be equal to one ($\theta =1$), which is the same as the default in the “R” program. The variable *X* in the Weibull distribution is expressed as $X \,{\sim }\,\mathscr {W}(\alpha ,\theta )$ or, $X \,{\sim }\,\mathscr {W}(\alpha , 1)$.

### Statistical measurements for assessment of results

The bias measure and standard deviation measure are considered in the sensitivity analysis indices for assessment of the robustness and quality of the gained results from Sobol method. The bias measure is given as,

24 $$\begin{aligned} \textrm{Bias}(T)=\mathbb {E}[T] - \epsilon , \end{aligned}$$

where *T* is an estimation of $\epsilon$, $\epsilon$ is a variable in a model, and $\mathbb {E}[T]$ is the mathematical expectation of *T*^32^. We have $\mathbb {E}[T]$ is the sum of all possible values from a random variable *X* and is as follows:

25 $$\begin{aligned} \mathbb {E}[T]=\int _{-\infty }^{\infty } X f(X)\,\textrm{d}X. \end{aligned}$$

Another assessment measure is known as standard deviation or standard error and is denoted by $\sigma$ where

26 $$\begin{aligned} \sigma = \sqrt{\frac{1}{n-1} \sum _{i=1}^n (X_i - \overline{X})^2}, \end{aligned}$$

where *X* is a random variable in the model, $\bar{X}$ is the mean of *X*, and *n* is the sample size of data.

In what follows, we analyse pressure, stresses and displacement (given in Section 2) using the Sobol method.

## Application of the Sobol method

The Sobol method evaluates the way in which the contribution of each input parameter affects the uncertainty of the output variables within the model framework. The dominance of contributions can be directly tied to individual input parameter or may emerge from higher-order interactions among the input parameters influencing the model’s output. The Sobol method takes into account two primary indices: the direct influence of uncertainty associated with each input parameter, or first-order index in (16), and the total Sobol index in (17). Furthermore, this method assesses the overall sum of uncertainty (direct and indirect contributions) for every input parameter in the model. The strategy to apply the Sobol method to the problem defined in Section 2 is described in the following five steps:

- The input parameters within the model are defined, and appropriate ranges are assigned to each parameter.
- Statistical simulations are conducted in the “R” program considering each input parameter, with a sample size of $N=20,000$, in the stress, pressure and displacement relations.
- Sensitivity indices of input parameters are calculated using the Saltelli-Janson estimators for first-order indices by means of (18) and for total indices by means of (19).
- The input parameters are then organized based on the highest values of the total indices (from higher values to lower values).
- Finally, assessment measures for the Sobol indices are performed, focusing on bias and standard deviation measures.

The “R” programming environment includes a feature for assessing Sobol sensitivity indices using a dummy parameter, which is computed for both first-order and total-order indices. This dummy parameter serves as a benchmark for identifying influential versus non-influential input factors in the model. If the Sobol index of any input parameter is lower than that of the dummy parameter, it indicates that the parameter does not significantly affect the model’s output. Furthermore, confidence intervals are estimated for each Sobol index, providing a measure of statistical reliability and allowing for robust interpretation of sensitivity results. The confidence interval is

27 $$\begin{aligned} CI=\overline{X}\pm z\frac{s}{\sqrt{N}}, \end{aligned}$$

where $\overline{X}$ is the sample mean, *z* is the confidence level value, *s* is the sample standard deviation, and *n* is the sample size. Table 1 depicts the range of each input parameter. The selected ranges are derived from common ranges that can be identified in porous bones^19–21^.

**Table 1:** Ranges of the input parameters for Bones^19–21^.

**Parameters**	**Possible Bone Ranges**
$$\alpha$$	(0, 1)
$P_0$	(0.05, 0.14kPa)
$\nu$	(0.2, 0.5) : cortical - 0.3, cancellous - 0.35
$\mu$	(0,12) : cortical - (4, 12GPa), Cancellous - (0.5, 1GPa)
*M*	(0, $\infty$)
$$t^*$$	(0.001, 1)
$$R^*$$	(1,5cm)

Table 2 presents the range and associated probability distribution for each input parameter considered in the model. Among these, Biot’s modulus and time exhibit a monotonic increasing behaviour within the scope of the bone analysis, as supported by the parameter relationships outlined in Table 1. Accordingly, we assign probability distributions that reflect this characteristic trend: the exponential distribution is selected for Biot’s modulus, while the Weibull distribution is chosen for time. These distributions align well with the physical behaviour and expected value ranges of the respective parameters, ensuring a realistic representation of uncertainty in the model.

**Table 2:** The ranges and probability distributions of the input parameters that are used in “R” program for the sensitivity analysis.

Parameters	under $\mathscr {U}$(p, q), $\exp$($\theta$), $\mathscr {W}$($\alpha$,1)
$\alpha$	$\mathscr {U}$(0, 1)
$P_0$	$\mathscr {U}$(0.05, 0.14)
$\nu$	$\mathscr {U}$(0.2, 0.5)
$\mu$	$\mathscr {U}$(0,12)
*M*	$\exp$ (1)
$t^*$	$\mathscr {W}$(0.001, 1)
$R^*$	$\mathscr {U}$(1,5)

We now apply the Sobol method to pressure, stresses and displacement, (5)- (9). First-order and total Sobol indices of each parameter in the expressions for pressure, stresses and displacement are illustrated in Table 3.

**Table 3:** Panel (a) gives the first-order index ($S_i$) and total Sobol index ($T_i$) for each input parameter, based on its corresponding range shown in Table 2, associated with pressure (Eq. (5)) and displacement (Eq. (9)). Panel (b) gives the first-order index ($S_i$) and total Sobol index ($T_i$) for each input parameter, based on its corresponding range shown in Table 2, associated with stress components ( Eqs. (6), (7) and (8)).

Sobol indices	*P*		$u_{r}$			
Type of index	$S_i$	$T_i$	$S_i$	$T_i$		
(a)						
$\alpha$	0.07432035	0.31069586	0.00036655	0.00982211		
$P_0$	0.03003938	0.08115344	0.04378677	0.09694226		
$\nu$	0.06379583	0.31958296	0.15378528	0.42059303		
$\mu$	0.00257845	0.12871030	0.13271951	0.36009938		
*M*	0.05508098	0.21043818	0.00040891	0.00999062		
$t^*$	0.15421784	0.44619788	0.19438654	0.51849268		
$R^*$	0.09628553	0.33589213	0.04990607	0.17446842		

Sobol indices	$\sigma _{rr}$		$\sigma _{\theta \theta }$		$\sigma _{zz}$	
Type of index	$S_i$	$T_i$	$S_i$	$T_i$	$S_i$	$T_i$
(b)						
$\alpha$	0.00012115	0.01500306	0.00846602	0.01499712	0.01217904	0.01966357
$P_0$	0.08916369	0.13014139	0.08848076	0.12930607	0.10340988	0.13765426
$\nu$	0.02064181	0.02470347	0.02136159	0.02192774	0.06852124	0.08795970
$\mu$	0.22488153	0.34860454	0.22588146	0.35571030	0.13453880	0.20470594
*M*	0.00016543	0.01492655	0.00838188	0.01459505	0.01139903	0.02079931
$t^*$	0.52387041	0.69513654	0.51540437	0.68726869	0.59990199	0.72625279
$R^*$	0.00004190	0.01516759	0.00876764	0.01498193	0.01101023	0.01815378

**Fig. 3:**
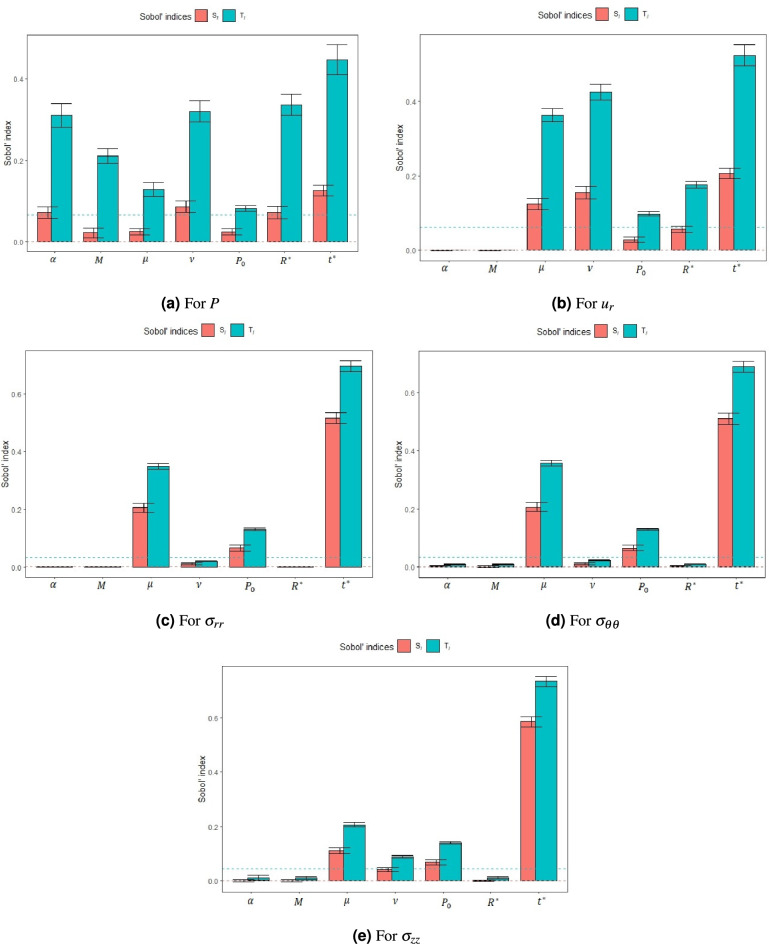
Sobol indices (first order and total order) of each input parameter associated with pressure, stress components and displacement for a poroelastic bone model under radial stress boundary conditions.

Figure 3 presents the Sobol sensitivity indices for each input parameter, with the first-order index ($S_i$) and total-order index ($T_i$) depicted in red and blue, respectively. The first-order index for the dummy parameter is shown as a dashed horizontal red line, while the corresponding total-order index is indicated by a dashed horizontal blue line. If the first order (total) Sobol index of any input parameter falls below the line representing the first order (total) dummy index, then, these inputs are indistinguishable from the dummy index (approximation error of the model) and, therefore, do not influence the model output. These benchmarks help distinguish influential parameters from non-influential ones. Confidence intervals for each Sobol index are represented by horizontal lines at the top of each bar, providing a measure of statistical reliability. In cases where computed indices are negative, the corresponding bars are truncated near zero, and negative values are not displayed, in accordance with standard visualisation practices. The order of input parameters based on the total Sobol index in each panel is summarised:

28 $$\begin{aligned} \begin{array}{lllllllllllllllllll} P:& T_{t^*}> T_{R^*}> T_{\nu }> T_{\alpha }> T_{M}> T_{\mu }> T_{P_0} ,\\ u_{r}:& T_{t^*}> T_{\nu }> T_{\mu }> T_{R^*}> T_{P_0}> T_{M}> T_{\alpha }\\ \sigma _{rr}:& T_{t^*}> T_{\mu }> T_{\nu }> T_{P_0}> T_{\alpha }> T_{M}> T_{R^*} ,\\ \sigma _{\theta \theta }:& T_{t^*}> T_{\mu }> T_{\nu }> T_{P_0}> T_{M}> T_{\alpha }> T_{R^*} ,\\ \sigma _{zz}:& T_{t^*}> T_{\nu }> T_{\mu }> T_{R^*}> T_{M}> T_{\alpha } > T_{P_0}. \end{array} \end{aligned}$$

It is clear that time, represented as $t^*$, is the most important (influential) parameter. The analytical solution is time-dependant and the sensitivity analysis reflects this fact. As this is the case for all the outputs, we turn our attention to analyse the other input parameters.

For the pressure, the nondimensionalised radius of the bone $R^*$ is the second most influential input parameter. We find that the Poisson ratio $\nu$ plays a greater role than the shear modulus $\mu$ on the pressure and the axial stress $\sigma _{zz}$, while the shear modulus $\mu$ is more influential than the other parameters on the radial $\sigma _{rr}$ and circumferential $\sigma _{\theta \theta }$ stresses. Indeed, all this has physical meaning for bones and it should be expected. On the other hand, we see that $R^*$, is the least influential factor on the stress components $\sigma _{rr}$ and $\sigma _{\theta \theta }$, while the initial pressure $P_0$ and the Biot’s coefficient $\alpha$ are the least influential parameters on the pressure, the stress component $\sigma _{zz}$ and the displacement $u_r$. In addition, the Poisson ratio $\nu$ plays a greater role than shear modulus $\mu$ on the pressure and axial stress response, while the shear modulus $\mu$ is more influential than the other parameters on the radial and circumferential stresses.

The assessment of Sobol indices is given by the measures of bias and standard deviation shown in Table 4 and Table 5, respectively, for all the model outputs. Bias and standard deviation values of both $S_i$ and $T_i$ for all the output models are very small, indicating the robustness of the Sobol indices that have been obtained.

**Table 4:** Panel (a) gives the bias measure of Sobol indices for pressure ( Eq. (5)) and the displacement (Eq. (9)), where the input parameters take the ranges detailed in Table 2. Panel (b) gives the bias measure of Sobol indices for stress components (Eqs. (6), (7), and (8)), where each input parameters takes the range detailed in Table 2.

Bias measures	*P*		$u_{r}$			
Type of index	$S_i$	$T_i$	$S_i$	$T_i$		
(a)						
$\alpha$	-0.0003226	-0.0005706	0.0019575	.0000068		
$P_0$	-0.0006045	0.0000980	0.0018783	-0.0001798		
$\nu$	0.0003506	-0.0013420	0.0015829	0.0004701		
$\mu$	0.0009159	-0.0009268	0.0013912	0.0003190		
*M*	0.0008693	-0.0007490	0.0020005	-0.0000007		
$t^*$	-0.0016799	0.0010324	0.0032773	0.0025315		
$R^*$	0.0007059	0.0002456	0.0010204	-0.0000052		

Bias measures	$\sigma _{rr}$		$\sigma _{\theta \theta }$		$\sigma _{zz}$	
Type of index	$S_i$	$T_i$	$S_i$	$T_i$	$S_i$	$T_i$
(b)						
$\alpha$	0.0008473	-0.0000013	0.0009381	0.0000541	0.0006410	-0.0003228
$P_0$	0.0025996	-0.0001983	0.0008645	0.0001476	-0.0002249	-0.0000962
$\nu$	0.0006230	-0.0000097	0.0011937	-0.0001081	0.0000651	0.0002940
$\mu$	-0.0001821	0.0008990	0.0006071	0.0003781	-0.0001994	-0.0005158
*M*	0.0008619	-0.0000031	0.0008535	-0.0000635	0.0001747	0.0000575
$t^*$	0.0004767	-0.0006099	-0.0000466	-0.0012133	0.0004070	0.0002439
$R^*$	0.0008581	-0.0000003	0.0016362	-0.0001378	-0.0004254	0.0001338

**Table 5:** In a parallel way to Table 4, now, this Table gives the standard deviation measures of Sobol indices.

Standard dev.	*P*		$u_{r}$			
type of index	$S_i$	$T_i$	$S_i$	$T_i$		
(a)						
$\alpha$	0.0354012	0.0153923	0.0151894	0.0000666		
$P_0$	0.0298850	0.0029644	0.0183613	0.0022937		
$\nu$	0.0348511	0.0104013	0.0205047	0.0099663		
$\mu$	0.0295866	0.0093056	0.0198608	0.0079185		
*M*	0.0343787	0.0087710	0.0152294	0.0000607		
$t^*$	0.0320637	0.0182711	0.0185492	0.0139642		
$R^*$	0.0342954	0.0100475	0.0189399	0.0046603		

Standard dev.	$\sigma _{rr}$		$\sigma _{\theta \theta }$		$\sigma _{zz}$	
type of index	$S_i$	$T_i$	$S_i$	$T_i$	$S_i$	$T_i$
(b)						
$\alpha$	0.0103969	0.0000213	0.0099026	0.0012400	0.0111479	0.0040588
$P_0$	0.0116144	0.0020679	0.0105122	0.0023352	0.0119738	0.0020922
$\nu$	0.0111602	0.0003809	0.0099214	0.0008263	0.0104449	0.0020464
$\mu$	0.0110759	0.0052274	0.0106843	0.0052385	0.0111140	0.0034991
*M*	0.0104029	0.00004735	0.0098156	0.0008209	0.0101565	0.0029198
$t^*$	0.0072997	0.0108550	0.0070518	0.0096885	0.0058643	0.0096442
$R^*$	0.01042494	0.0000106	0.0098054	0.0007423	0.0099252	0.00239167

**Fig. 4:**
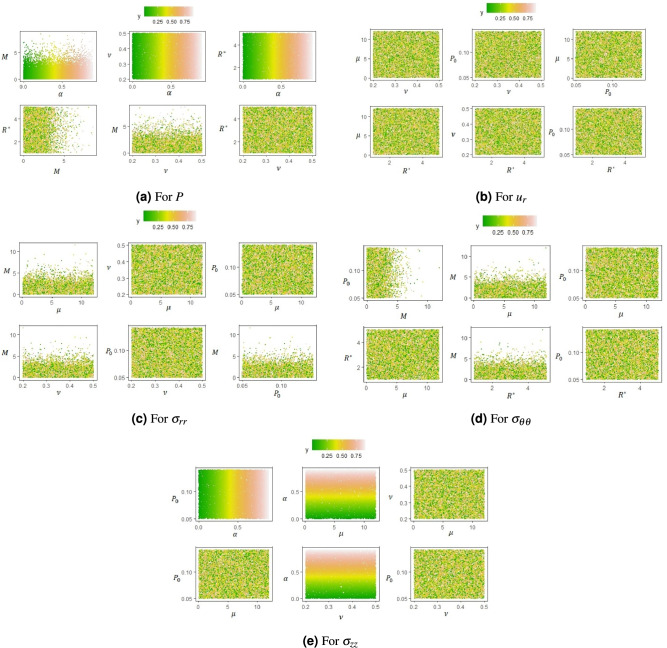
Second-order interaction effects of input parameters on pressure, panel (**a**), displacement, panel (**b**) and stress components, panels (**c**–**e**), are given.

For completeness, we consider the interaction effects among the input parameters (excluding time). Second order indices capture interaction effects between two input parameters on the output of the model. The results of this analysis are shown in the scatter plots of Fig. 4 in which a particular colour is a visual representation of the value of the the model output, denoted by variable *y*. Green colour (dots) represents a low output value and as this colour changes from green to red the model output increases its value from low (green) to high (red). In each scatter plot the colour of the model output varies with the two parameters indicated on the axes.

Figure 4 shows a broad spectrum of results and we now discuss them. Recall that $t^*$ has a substantial first-order effect on all the model outputs, which leads to minimal interaction with the other input parameters. Therefore, $t^*$ is disregarded in this joint interaction assessment. Higher-order interaction effects on the pressure, the stress components and the displacements are depicted in a multi-scatter plot for $\alpha$, $\mu$, $\nu$, *M*, $R^*$, and $P_0$.

In Figure 4a the presence of significant vertical stripes and alignment of green, yellow and red dots between $\alpha$ and $\nu$, as well as $\alpha$ with $R^*$ and *M* for the pressure output is noteworthy. For small values of *M*, $\nu$ and $R^*$ (vertical axis in the scatter plots), it is the value of $\alpha$ (horizontal axis) that dictates whether the output pressure is low or high. We also note that joint interactions between the Biot’s Modulus *M* and the $\alpha$ show a pattern of dots not appearing beyond the value of 5, which can be explained by the nature of the Biot’s modulus and the fact that it tends to $\infty$.

Similarly, in panel Fig. 4e for the stress component $\sigma _{zz}$ there are significant green, yellow and red dots aligned as stripes between $\alpha$ and $\nu$, as well as $\alpha$ with $P_0$ and $\mu$. Between $\alpha$ and $P_0$ we see vertical stripes. This means that when there are low input values of $P_0$ (vertical axis), then it is the value of $\alpha$ (horizontal axis) that dictates whether the output longitudinal stress $\sigma _{zz}$ is low or high. When we consider the relationships between $\alpha$ and $\mu$ as well as with $\nu$ we see horizontal stripes of green, yellow and red dots. This means that when we have any value of $\mu$ and $\nu$ it is the value of $\alpha$ the one that dictates whether the output (longitudinal stress) is high or low.

These patterns reflect that there are higher-order interaction between each of these sets of two input parameters. This finding underscores the importance of focusing on the total Sobol index for these parameters in the model. This emphasis is warranted due to the anticipated higher joint effect among these input parameters. To highlight further this result, one can note that for these parameters the values of their first-order indices are considerably lower than their total Sobol indices.

Random scattering of different colour dots in other panels is associated with no significant joint interactions. The resulting colour mix does not reveal any systematic pattern, indicating a limited higher-order interaction effect between these parameters on the model output. Therefore, for the displacement and the stress components $\sigma _{rr}$ and $\sigma _{\theta \theta }$ none of the input parameters have any joint interaction effects on the output model. In these cases, the first-order index, $S_i$, associated with these input parameters, is sufficient to quantify the sensitivity of the input parameter on the model output.

## Conclusions

In this work, we use the analytical solution for the equations that describe the mechanical behaviour of a porous material as it is presented in Asghari et al. (2024)^37^. We investigate a solid cylinder with a microstructure comprising a porous elastic matrix, which is assumed to be isotropic and representing the human bone as an example of hard mechanical systems. We then investigate the physical quantities that our solution provides (pressure, stresses and displacement) for the bone via a sensitivity analysis on the input parameters. This allows us to to determine the influence of the several underlying input parameters on each individual output.

The analysis captures that time $t^*$ is the most influential parameters on all the outputs, i.e., pressure, stress components and displacement. We find that the Poisson ratio $\nu$ plays a greater role than shear $\mu$ on pressure and longitudinal stress, and the shear modulus $\mu$ is more influential than the other parameters on the radial and circumferential stresses. Furthermore, the role of the Biot’s coefficient $\alpha$ on pressure is fully appreciated via the joint second-order interactions with the Poisson ratio $\nu$ and the non-dimensionalised radius $R^*$ of the bone (cylinder), where interstitial pressure is a key value associated with bone remodelling and fracture healing. Similarly the role of the Biot’s coefficient $\alpha$ on the longitudinal stress $\sigma _{zz}$ is fully appreciated via the joint second-order interactions with the shear $\mu$, the Poisson ratio $\nu$, and the initial pressure $P_0$.

The prominence of interstitial pressure as a sensitive output, especially through interactions involving the Biot’s coefficient $\alpha$, Poisson ratio $\nu$ and non-dimensional radius $R^*$ highlights its vital role in mechanotransduction, a key driver of bone remodelling. The strong influence of time $t^*$ on all outputs suggests that transient fluid-solid interactions are critical in capturing the dynamic environment that the osteocyte cells respond to during remodelling and healing. The joint sensitivity of longitudinal stress $\sigma _{zz}$ to $\alpha$, $\mu$, $\nu$, and initial pressure $P_0$ reflects the complex mechanical stimuli that regulate remodelling in load-bearing regions, such as the in the shaft of long bones. These findings support the hypothesis that fluid pressure gradients, modulated by poroelastic parameters, act as biochemical triggers for cellular activity, influencing both bone formation and resorption.

Sobol sensitivity analysis has been used to verify Biot’s poroelastic model for bones by assessing how input parameters, such as elastic moduli and poroelastic moduli, influence key outputs such as stresses, displacement and pressure. By computing first-order and total-order Sobol indices, one can confirm that the model responds in line with physical expectations, such as fluid-related parameters (e.g., $\alpha$, *M*, $\nu$) dominate pressure sensitivity. This technique plays a crucial role in detecting implementation errors and validating the parameter scalings that we have chosen, such as displacement being non-dimensionalised by bone radius and the stress by initial pressure.

We note that the sensitivity analysis utilizes a collection of input parameters presented in Table 1. These parameters have been selected via a random sampling methodology and are associated with poroelastic bone modelling. The modelling of bone structures has been executed via a poroelastic approach in many previous works^5,23,24^. The pores within bones are filled with substances such as bone marrow, blood, interstitial fluid, and cells making this a suitable application for the model proposed herein and providing an understanding of critical parameters^24,25^.

The current study is subject to some limitations and extensions that can increase the applicability of results. The governing poroelastic equations from which we begin our analysis were proposed by Biot following experimental development and describe the material on the macroscale. It has been proved by various authors that it is possible to obtain the same governing equations of a poroelastic material via the use of homogenization techniques^46,59,60^. These techniques develop the macroscale equations via the upscaling of microscale problems that are proposed to capture the interactions of phases on a repeating unit of the microstructure of the material. Models of this kind have been developed and can encode a variety of different structural features such as pore geometry and arrangement or even additional elastic or porous phases in the effective coefficients^61–63^. The effective coefficients include the effective elasticity tensor that contributes to the stresses from which the analytical solution is obtained. Therefore we would be able to encode this extra detail from the microstructure in the solution equations on which we carry out the sensitivity analysis. In this work the effective elasticity tensor describing our cylindrical porous material is assumed to be isotropic. If we determine our macroscale governing equations for the poroelastic material via homogenization techniques, then we could obtain more complicated symmetries such as cubic, tetragonal or orthogonal ones. These symmetries would be incorporated into the analytic solution. The different geometries could be more realistic of complex hard hierarchical materials beyond the bone example given here. Both of these extensions would provide a detailed picture of bone modelling and the important parameters.

The parameter ranges are currently obtained from sources in the literature, nevertheless, one would be able to inform the parameter ranges via micromechanical simulations. This would rely upon using the governing equations of poroelastic materials that were obtained via application of the asymptotic homogenization technique^46,59,60^. This would mean that we would have a multi-scale model for the bones where the coefficients/parameters of the governing equations are calculated via the solution of porescale differential problems^64–66^. The result of the porescale differential problems would provide a range for each of the parameters that could be used within the sensitivity analysis and would incorporate the porescale geometry and mechanical properties in the parameter ranges, creating more accurate results from the Sobol sensitivity analysis.

Future sensitivity analyses may be conducted to model complex materials for instance featuring instability modes (including necking, bending, beading, helical buckling, bulging, and prismatic bifurcation)^67–72^, as well as changes in the material properties, for example as a result of remodelling and growth^73–76^.

## Data Availability

All data generated or analysed during this study are included in this published article.
